# From Euphoria to Emergency: Exploring the Role of K2/Spice in Diffuse Alveolar Hemorrhage

**DOI:** 10.7759/cureus.41887

**Published:** 2023-07-14

**Authors:** Nishant Allena, Laura Yapor, Muhammad Yasir Anwar, Trupti Vakde

**Affiliations:** 1 Internal Medicine, BronxCare Health System, Bronx, USA; 2 Pulmonary and Critical Care, BronxCare Health System, Bronx, USA

**Keywords:** acute respiratory distress syndrome (ards), lung injury, cannabis use, synthetic cannabis, e-cigarette and vaping product use associated lung injury (evali), hemoptysis, bilateral pulmonary infiltrates, k2/spice, synthetic cannabinoids, diffuse alveolar hemorrhage

## Abstract

Marijuana or cannabis has been one of the most widely used recreational drugs, in the United States. However, a sinister counterpart has emerged in recent times: K2/Spice, a synthetic rendition of tetrahydrocannabinol (THC), capturing increasing popularity. Alarming reports have linked this synthetic compound to a multitude of life-threatening complications, ranging from acute kidney injury (AKI) from direct nephrotoxicity to cardiac arrest. Here we present the case of a 34-year-old man who presented with hemoptysis, later found to have diffuse alveolar hemorrhage (DAH) on the investigation after smoking K2/Spice successfully treated with a course of intravenous steroids. The case presented underscores the urgent need for increased awareness about the potential complications associated with synthetic compounds like K2/Spice, such as diffuse alveolar hemorrhage, and the importance of developing effective treatment strategies.

## Introduction

As marijuana gains a growing user base, thanks to its legalization for recreational purposes in 23 states across the United States [1], an ominous threat lurks in the shadows: synthetic cannabinoids infused with natural counterparts. Among these, K2/Spice, a synthetic rendition of tetrahydrocannabinol (THC), the psychoactive compound in marijuana, has witnessed a surge in usage. However, the true extent of its side effects continues to unravel, with a constellation of risks including psychosis, hypertension, coronary vasospasm, and the rare yet life-threatening complication of diffuse alveolar hemorrhage (DAH). Given the nature of synthetic cannabinoids to go undetected on standard urine toxicology panels and its catastrophic complications, we aim to shed light on the extraordinary occurrence of synthetic marijuana-induced DAH, highlighting the urgency for understanding and addressing this dangerous phenomenon.

## Case presentation

**Figure 1:**
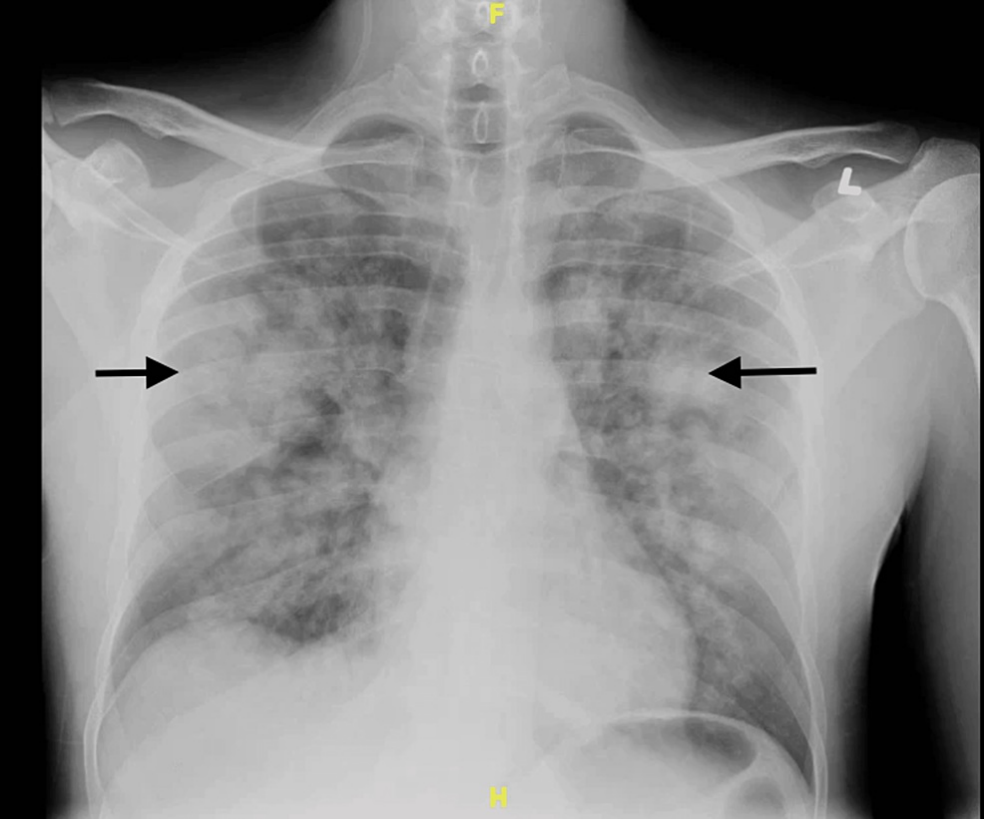
X-ray chest AP view of the chest showing areas of consolidation. Black arrows showing areas of consolidation.

A 34-year-old male with a past medical history of asthma, hypertension, sickle cell trait, alcohol use disorder, and self-reported K2/Spice use presented to the ED with a complaint of hemoptysis for one day. He also complained of a yellow productive cough and chest pain for four days. His SpO_2_ was 87% to 89% in room air, and he was placed on 4 liters/min of supplemental oxygen via a nasal cannula. A chest x-ray showed multiple ill-defined pulmonary masses and areas of consolidation (Figure 1).

**Figure 2:**
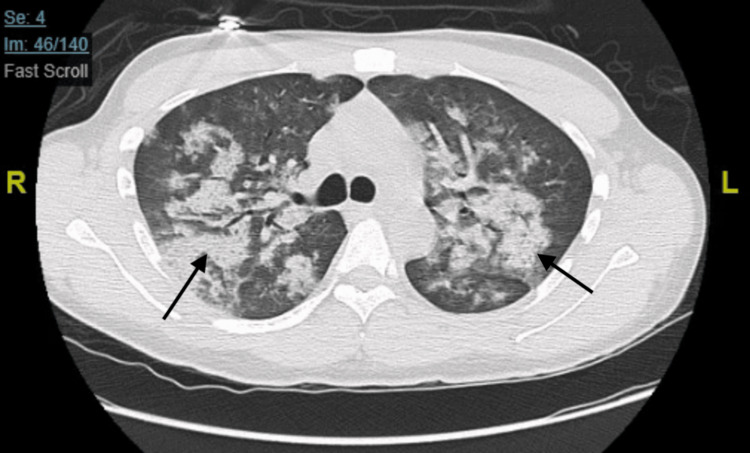
CT chest with contrast showing multiple diffuse ground-glass opacities. Black arrows showing ground-glass opacities.

This was followed by a CT scan with contrast, which showed multiple ground-glass opacities present throughout both lungs (Figure 2).

Labs revealed a hemoglobin of 13.8 mg/dL and a platelet count of 260 k/uL. The white blood count was 22k/µL, the neutrophil percentage of 93.3, the lymphocyte percentage of 2.2%, the eosinophil percentage of 0.1, the prothrombin time (PT) was 13.6 seconds, the INR of 1.14, and the activated partial thromboplastin time (APTT) was 34.4 seconds. On day six, the hemoglobin was 12.7 g/dL, the WBC count was 9.9, the neutrophil percentage of 73.1, the lymphocyte percentage of 5.7%, and the eosinophil percentage of 0.1%.

**Figure 3:**
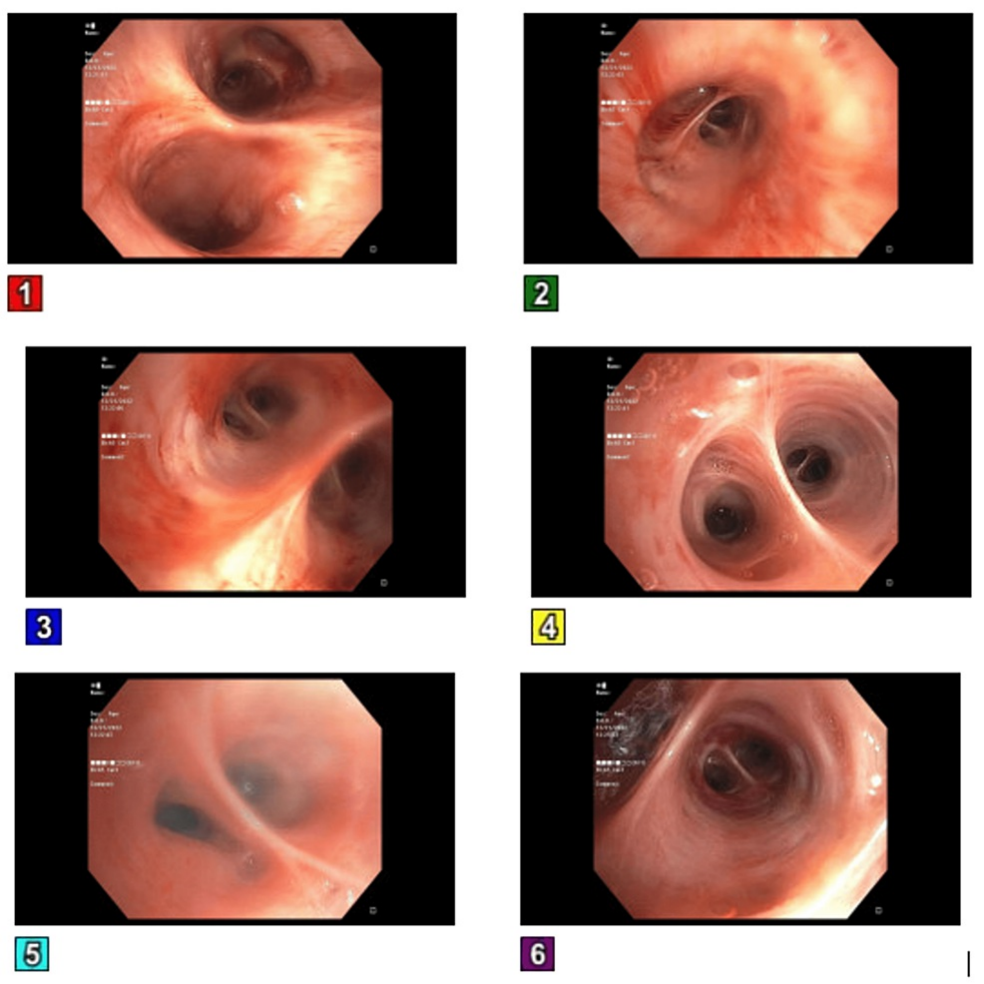
Bronchoscopy showing no active source of bleeding and mucoid serosanguineous secretions. Panel 1: Trachea, Panel 2: Right upper lobe, Panel 3: Right middle lobe, Panel 4: Right lower lobe, Panel 5: Left mainstem bronchus, and Panel 6: Left lower lobe.

The patient was subsequently transferred to the intensive care unit (ICU) due to refractory hypoxic respiratory failure, necessitating intubation, as the administration of oxygen via nasal cannula proved ineffective in alleviating the persistent hypoxia and the patient displayed tachypnea. A bronchoscopy was done, which showed no active source of bleeding, and mucoid serosanguineous secretions were noted (Figure 3).

**Figure 4:**
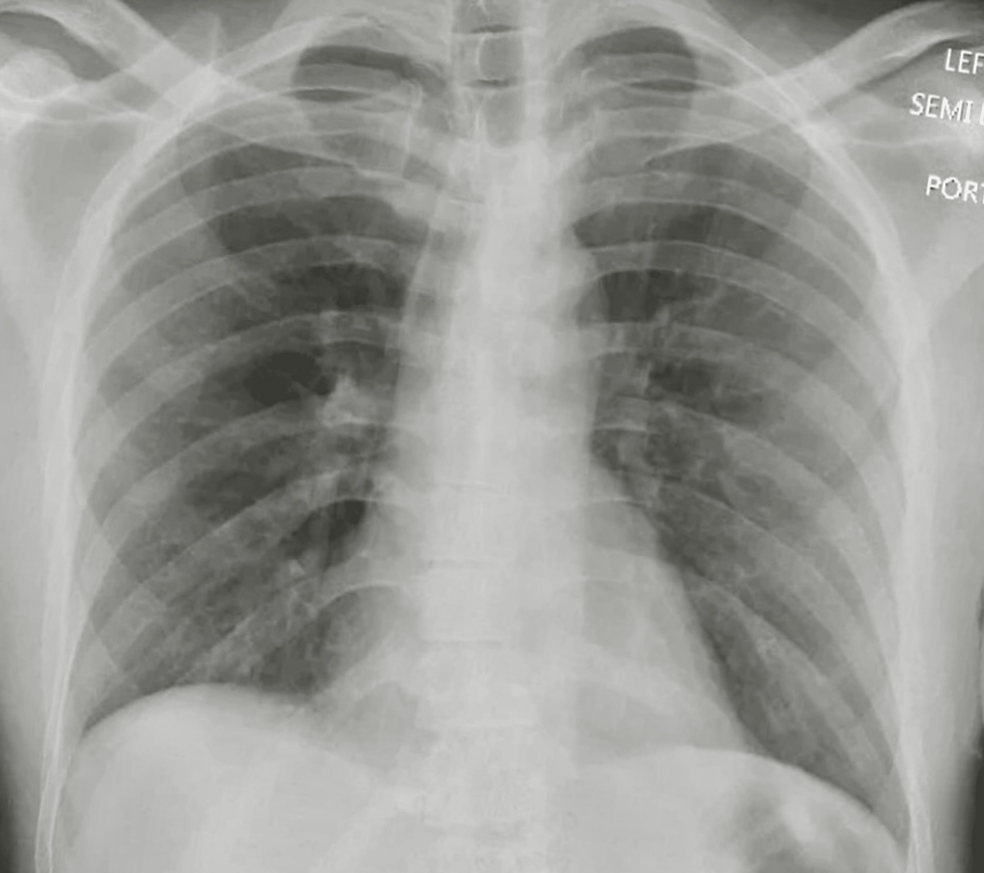
X-ray chest on day six of admission showing resolution of bilateral opacities.

He was started on methylprednisolone IV 250 mg every six hours as the suspicion for a vaping-associated lung injury (VALI) was high. Empiric IV antibiotics were started to cover for any underlying pneumonia, and vancomycin 1 g every 12 hours, IV azithromycin 500 mg OD, and aztreonam 1 g every eight hours in view of the patients' penicillin allergy were administered. An autoimmune pathology was next on the differential, with possible causes being either primary small vessel vasculitis, primary immune complex-mediated vasculitis, or secondary vasculitis. Hence a vasculitis workup that included glomerular basement membrane (GBM) antibody, antineutrophilic cytoplasmic antibody (ANCA), rheumatoid factor, cyclic citrullinated peptide (CCP) antibody (IgG), antibody to anti scleroderma-70, anti-nuclear antibody, DNA antibody, antibody to Jo-1, and antiphospholipid antibody was done, which came back negative, and IV methylprednisolone was tapered to 62.5 mg every eight hours for four days, followed by 62.5 mg every 12 hours for one more day, and then discontinued, completing a total course of six days. Urine toxicology was also unrevealing, but testing for synthetic cannabinoids could not be performed. Mycobacteria acid-fast bacilli stain was also negative. A repeat chest x-ray done on day six of admission showed an interval resolution of diffuse bilateral airspace opacities (Figure 4), following which the patient was discharged home on no supplemental oxygen. The patient was later followed up in our pulmonary clinic, where he presented in his usual state of health.

## Discussion

Diffuse alveolar hemorrhage (DAH) is a serious medical condition that causes capillaritis and inflammation in the alveoli, which can lead to respiratory failure, hemoptysis, anemia, and diffuse alveolar infiltrates. It is primarily associated with systemic autoimmune diseases such as anti-neutrophil cytoplasmic antibody-associated vasculitis, anti-glomerular basement membrane disease, and systemic lupus erythematosus (SLE). Other causes include inhalation of toxins, drug use, and coagulation disorders. Pulmonary capillaritis, which presents with neutrophilic infiltrates in the interstitium, is the most common form of DAH [2].

In New York State, patients primarily in the age group of 14 to 71 years old have developed vaping-induced lung injury, with 62% being under the age of 25 [3]. From those patients developing any kind of vaping-induced lung injury, 91% had consumed tetrahydrocannabinol (THC) containing products [4]. Synthetic cannabinoids like "K2-Spice" have been shown to be 30-100-fold more potent than Δ9-tetrahydrocannabinol (THC), these are believed to bind to cannabinoid receptor 1 (CB1R). The high potency of this cannabinoid leads to increased use in young adults. Overstimulation of CB1R in the lungs has been linked to lung injury, alveolar inflammation, and fibrosis [5]. 

The treatment approach for DAH involves three main categories: hemodynamic stabilization and airway protection, addressing the underlying cause, and achieving rapid local hemostasis. Prompt intubation and ventilation are necessary to manage the life-threatening hypoxic respiratory failure associated with DAH. Ventilation strategies depend on the presence of acute respiratory distress syndrome (ARDS), with high positive end-expiratory pressure (PEEP) strategies recommended according to ARDS guidelines [6]. 

The appropriate treatment for diffuse alveolar hemorrhage (DAH) depends on the underlying cause. Corticosteroids have long been established as the standard treatment for DAH due to their ability to mitigate acute inflammatory responses. In cases where DAH is attributed to toxin exposure, the primary therapeutic approach involves discontinuation or withdrawal of the offending toxin [7].

In the context of ANCA-associated vasculitis (AAV), which can give rise to DAH, a recommended treatment strategy entails high-dose pulse steroid therapy. This entails administering a daily dosage of 500 to 1,000 mg of steroids for a period of three days, followed by the initiation of oral prednisone at a daily dose of 1 mg/kg (not exceeding 60 to 80 mg) for a duration of two to four weeks. Subsequently, a gradual tapering of prednisone dosage is commenced [8].

While the available literature on K2-induced DAH is limited to a few case reports, there have been reports of favorable outcomes when corticosteroids were employed as part of the treatment regimen. The chosen treatment approach by Imtiaz et al. involved administering high-dose steroids for a period of three days, after which the patient was transitioned to a daily dose of 40 mg of prednisone. Over the course of four days, the prednisone dosage was gradually tapered down to 10 mg per day, and subsequently, the medication was completely discontinued [9]. Another case report by Adelman et al. also employed methylprednisolone as a part of their treatment strategy [10].

The prognosis of K2-Spice-induced diffuse alveolar hemorrhage (DAH) cannot be ideally predicted based on the published literature owing to the very limited number of cases reported. There have been published cases of coagulopathies seen in patients using K2-Spice. This coagulopathy is attributed to the lacing of K2-Spice with long-acting anticoagulant compounds. There has been an outbreak of coagulopathy associated with K2-Spice use in Illinois in 2018 leading to hemorrhage in multiple organs and fatalities [11].

In our case, we are limited by the inability to confirm the presence of tetrahydrocannabinol (THC), but taking into account the negative findings of the vasculitis workup and the favorable response to steroid treatment, we strongly posit K2/Spice as the primary etiological factor responsible for diffuse alveolar hemorrhage (DAH) in our patient.

In general, the prognosis for K2-Spice-induced DAH is better with prompt intervention and the avoidance of further exposure to the drug.

## Conclusions

The emergence of synthetic marijuana, including substances like K2/Spice, as a potential cause of diffuse alveolar hemorrhage (DAH) warrants significant attention. While DAH is already recognized as a rare yet life-threatening complication, the inclusion of synthetic marijuana as a novel causative factor highlights the need for careful management in a closely monitored medical setting.

Given the potency of synthetic cannabinoids, lack of detection on regular urine toxicology panels and their potential to overstimulate the cannabinoid receptors in the lungs, resulting in lung injury, inflammation, and bleeding, the recognition of synthetic marijuana-induced DAH is crucial. With prompt diagnosis and implementing appropriate management approaches, healthcare providers can enhance patient outcomes and promote safer practices regarding substance use.
